# l-Cystathionine Inhibits the Mitochondria-Mediated Macrophage Apoptosis Induced by Oxidized Low Density Lipoprotein

**DOI:** 10.3390/ijms151223059

**Published:** 2014-12-11

**Authors:** Mingzhu Zhu, Junbao Du, Siyao Chen, Angie Dong Liu, Lukas Holmberg, Yonghong Chen, Chunyu Zhang, Chaoshu Tang, Hongfang Jin

**Affiliations:** 1 Department of Pediatrics, Peking University First Hospital, Beijing 100034, China; E-Mails: zhumingzhu1988521@126.com (M.Z.); junbaodu1@126.com (J.D.); 15201286434@163.com (S.C.); chenyh3094@sina.com (Y.C.); chunyu5781@163.com (C.Z.); 2 Key Laboratory of Molecular Cardiology, Ministry of Education, Beijing 100191, China; E-Mail: tangchaoshu@263.net.cn; 3 Department of Medical and Health Sciences, Linköping University, Linköping 58183, Sweden; E-Mails: angie.dongliu@gmail.com (A.D.L.); lucas.holmberg20@gmail.com (L.H.); 4 Department of Physiology and Pathophysiology, Peking University Health Science Center, Beijing 100191, China

**Keywords:** l-cystathionine, ox-LDL, macrophage, mitochondrial, oxidative stress, apoptosis, caspase-9, caspase-3

## Abstract

This study was designed to investigate the regulatory role of l-cystathionine in human macrophage apoptosis induced by oxidized low density lipoprotein (ox-LDL) and its possible mechanisms. THP-1 cells were induced with phorbol 12-myristate 13-acetate (PMA) and differentiated into macrophages. Macrophages were incubated with ox-LDL after pretreatment with l-cystathionine. Superoxide anion, apoptosis, mitochondrial membrane potential, and mitochondrial permeability transition pore (MPTP) opening were examined. Caspase-9 activities and expression of cleaved caspase-3 were measured. The results showed that compared with control group, ox-LDL treatment significantly promoted superoxide anion generation, release of cytochrome c (cytc) from mitochondrion into cytoplasm, caspase-9 activities, cleavage of caspase-3, and cell apoptosis, in addition to reduced mitochondrial membrane potential as well as increased MPTP opening. However, 0.3 and 1.0 mmol/L l-cystathionine significantly reduced superoxide anion generation, increased mitochondrial membrane potential, and markedly decreased MPTP opening in ox-LDL + l-cystathionine macrophages. Moreover, compared to ox-LDL treated-cells, release of cytc from mitochondrion into cytoplasm, caspase-9 activities, cleavage of caspase-3, and apoptosis levels in l-cystathionine pretreated cells were profoundly attenuated. Taken together, our results suggested that l-cystathionine could antagonize mitochondria-mediated human macrophage apoptosis induced by ox-LDL via inhibition of cytc release and caspase activation.

## 1. Introduction

Atherosclerosis (AS) is a common cardiovascular disease with high incidence in the global population, which has become a global health burden. Nevertheless, the pathogenesis of AS has not been fully understood. Monocyte-macrophage plays an essential role in the development of AS [1]. Once the vascular endothelium is damaged, the low density lipoprotein (LDL) in blood invades vascular intima and become oxidized to ox-LDL, which can be phagocytized by monocyte/macrophages to form foam cells. Macrophage-derived foam cell promotes the formation of atherosclerotic plaque, eventually leading to atherosclerotic lesions [2,3,4]. Macrophage apoptosis is closely associated with the instability of atherosclerotic plaque, which is an important factor in the late atherosclerotic lesions. Macrophage apoptosis results in failed phagocytosis of apoptotic smooth muscle cells and macrophages, promoting the formation and enlargement of lipid core. In addition, the apoptotic macrophages rich of free cholesterol could damage fibrous cap by releasing matrix degradation proteases and generating inflammatory cytokines such as TNF-α and IL-1β to cause secondary necrosis and pro-inflammatory responses, thus leading to the plaque instability. Therefore, inhibiting macrophage apoptosis is of great significance to prevention of AS process and cardiovascular diseases [5].

The mechanisms responsible for the macrophage apoptosis in AS are in need of being fully explored. It is generally believed that the regulatory mechanisms for macrophage apoptosis are mainly through extrinsic death receptor pathway, intrinsic mitochondria pathway including activation of cytochrome c (cytc) and caspase, and endoplasmic reticulum stress (ERS) apoptosis pathway [6]. Mitochondrion is the control center of the cell life activities as well as the control center of apoptosis [7], which plays an important role in cell apoptosis [8]. Oxidative stress is the pathogenic factor of many cardiovascular diseases, and can induce apoptosis. In recent years, more and more studies have shown that oxidative stress can lead to apoptosis through mitochondria-mediated pathways [9,10,11].

l-cystathionine is a non-protein thioether containing amino acids, mainly produced in the metabolic transformation process of methionine to cysteine in the body [12]. So far, we know little about the relatively independent biological effects of l-cystathionine, in addition to its important role in sulfur transformation process as the key intermediate [13,14]. Preliminary studies have shown that l-cystathionine plays an important regulatory role in such processes as superoxide radicals scavenging [15,16], liver protection [17,18] and endoplasmic reticulum stress [19], and can inhibit the apoptosis of U937 and HepG2 cells through preventing the excretion of glutathione [20]. Nevertheless, how l-cystathionine regulates macrophage mitochondria-related apoptosis remains unknown.

Therefore, in the present study, we explored the impact of l-cystathionine on oxidative stress and apoptosis in human macrophages induced by ox-LDL and the possible mechanisms.

## 2. Results

### 2.1. l-Cystathionine Inhibited Oxidative Stress Induced by ox-LDL in Human Macrophages

**Figure 1:**
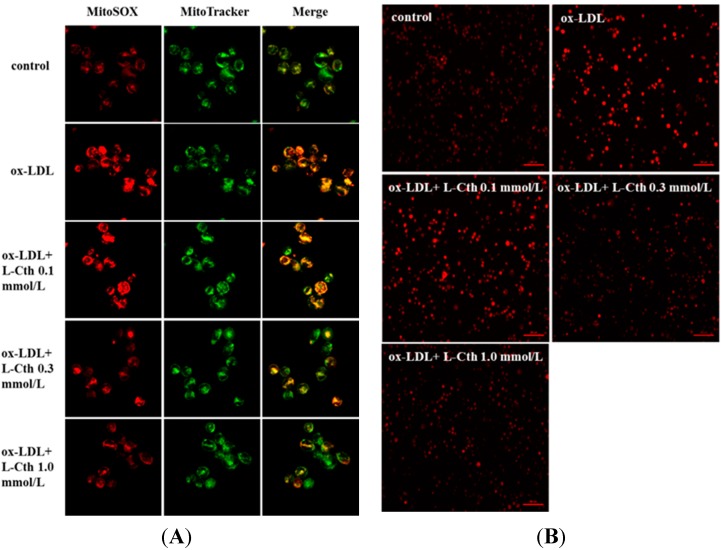
Changes of superoxide generation in human macrophages. (**A**) Mitochondrial superoxide generation in human macrophages detected by MitoSOX, with red fluorescence indicating mitochondrial superoxide and green indicating mitochondria; (**B**) Superoxide anion in human macrophages examined by DHE. l-Cth: l-cystathionine.

Firstly, to explore the impact of l-cystathionine on mitochondrial oxidative stress in human macrophages, MitoSOX reagent was used to detect mitochondrial superoxide generation. Results showed that mitochondrial superoxide generation in the ox-LDL group was significantly increased compared with the control group. Pretreatment with 0.1 mmol/L l-cystathionine did not change mitochondrial superoxide generation. However, with pretreatment of 0.3 and 1.0 mmol/L l-cystathionine, the generation of mitochondrial superoxide dramatically declined (Figure 1A).

Then, DHE probe was used to examine superoxide anion production in human macrophages. Results demonstrated profoundly increased superoxide anion production after ox-LDL treatment. Superoxide was not reduced by 0.1 mmol/L l-cystathionine, whereas with 0.3 and 1.0 mmol/L l-cystathionine administration, superoxide anion production was significantly decreased (Figure 1B).

### 2.2. l-Cystathionine Reversed the Inhibition of Mitochondrial Membrane Potential Induced by ox-LDL in Human Macrophages

**Figure 2:**
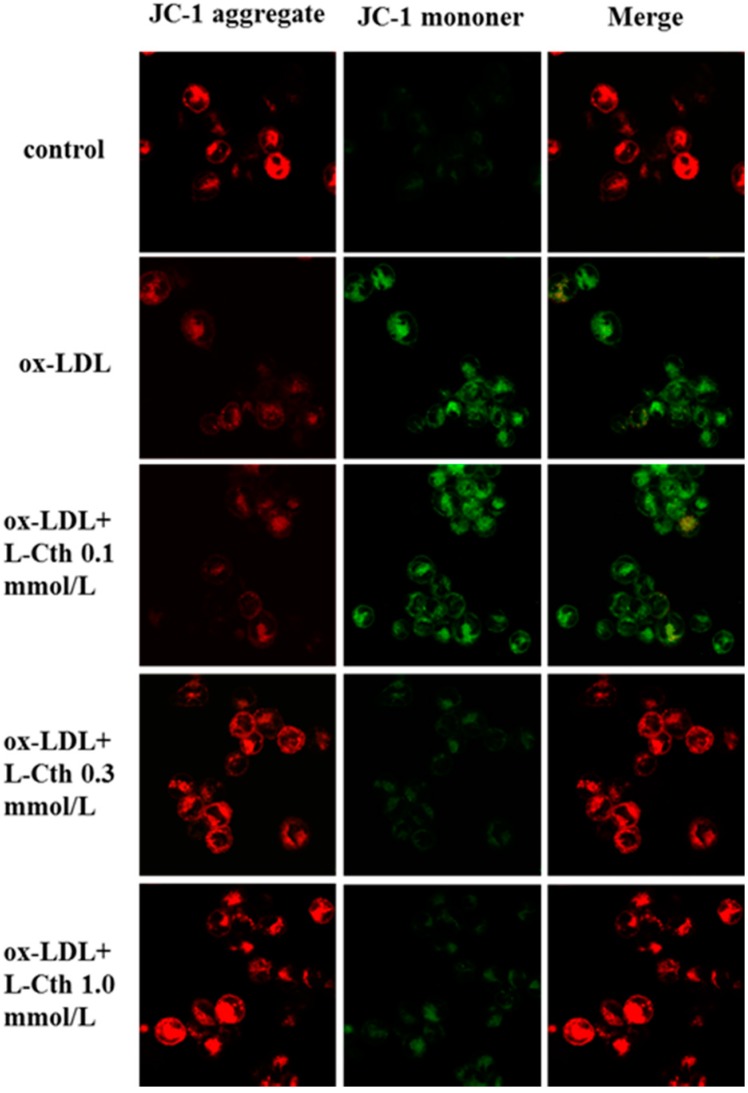
Changes in mitochondrial membrane potential in human macrophages, with red fluorescence indicating high mitochondrial membrane potential, and green low mitochondrial membrane potential. l-Cth: l-cystathionine.

JC-1 was used for measurement of mitochondrial membrane potential in human macrophages. Results showed that ox-LDL markedly reduced mitochondrial membrane potential compared to the control group. No significant effect of 0.1 mmol/L l-cystathionine on mitochondrial membrane potential was observed, while mitochondrial membrane potential was markedly increased after pretreatment with 0.3 and 1.0 mmol/L l-cystathionine (Figure 2).

### 2.3. l-Cystathionine Antagonized Mitochondrial Permeability Transition Pore (MPTP) Opening Induced by ox-LDL in Human Macrophages

**Figure 3:**
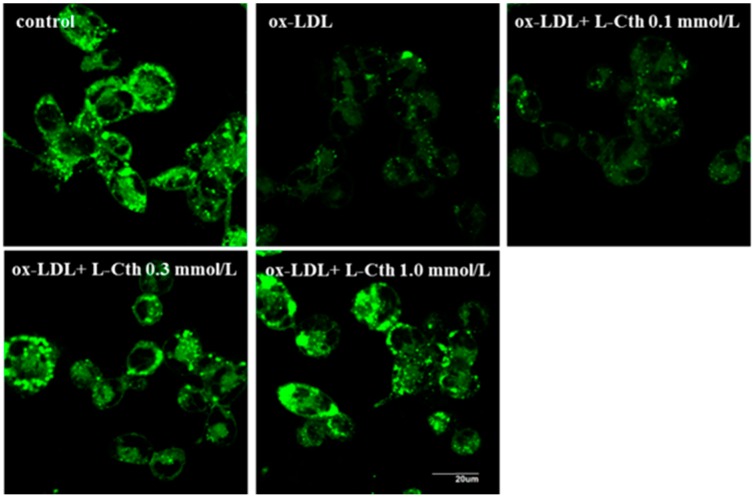
Changes in Mitochondrial Permeability Transition Pore (MPTP) opening in human macrophages. l- Cth: l-cystathionine.

MPTP opening in human macrophages was measured using fluorescence assay. Compared with control group, fluorescence intensity was significantly weakened in ox-LDL group, suggesting increased MPTP opening. When human macrophage was pretreated with 0.1 mmol/L l-cystathionine, fluorescence intensity did not alter. However, significantly enhanced fluorescence intensity was observed when pretreated with 0.3 and 1.0 mmol/L l-cystathionine, implying the inhibition of MPTP opening (Figure 3).

### 2.4. l-Cystathionine Inhibited the Release of Cytc from the Mitochondrion into the Cytoplasm Induced by ox-LDL in Human Macrophages

**Figure 4:**
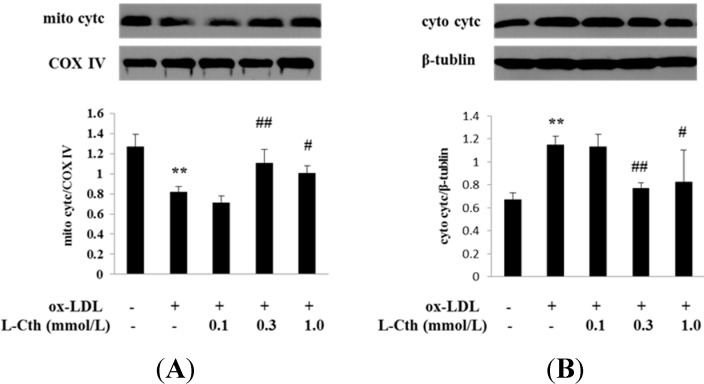
Cytcprotein expression in mitochondrion and cytoplasm in human macrophages. (**A**) Cytc protein expression in mitochondrionin human macrophages; (**B**) Cytc protein expression in cytoplasm in human macrophages. l-Cth: l-cystathionine; ** *p* < 0.01 compared with control group; ^##^ *p* < 0.01 compared with ox-LDL group; ^#^ *p* < 0.05 compared with ox-LDL group. Data are presented as mean ± SD of three independent experiments performed in triplicate.

Cytc protein expression in mitochondrion and cytoplasm in human macrophages was detected by western blot. Data showed a significantly downregulated mitochondrial cytc expression along with upregulated cytoplasmic cytc expression in ox-LDL group compared to that of control group, implying the release of cytc from mitochondrion into cytoplasm. Pretreatment with 0.1 mmol/L l-cystathionine had no effect on the cytc expression in mitochondrion and cytoplasm. Interestingly, 0.3 and 1.0 mmol/L l-cystathionine significantly upregulated cytc expression in mitochondrion but downregulated cytc expression in cytoplasm, demonstrating an inhibiting effect on the release of cytc from mitochondrion into cytoplasm (Figure 4A,B).

### 2.5. l-Cystathionine Suppressed Cell Apoptosis Induced by ox-LDL in Human Macrophages

**Figure 5:**
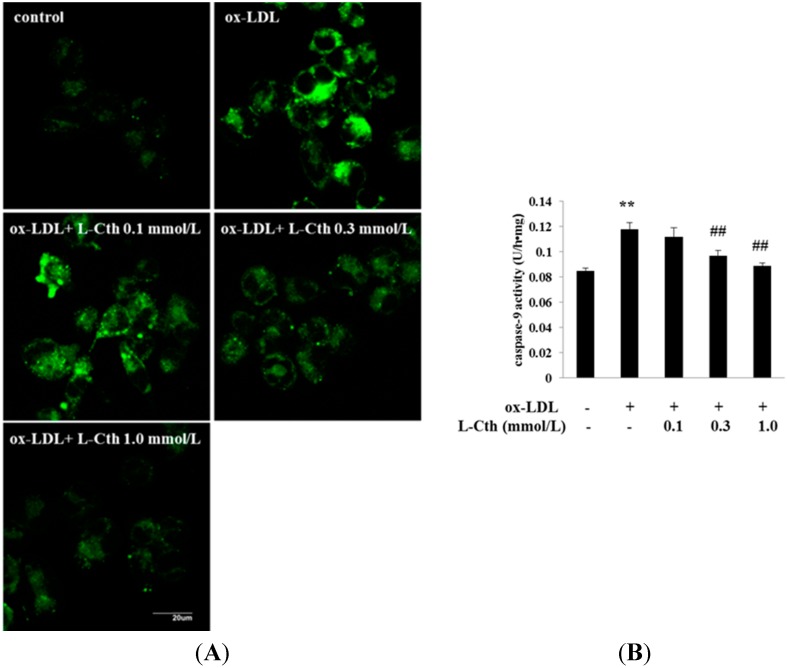

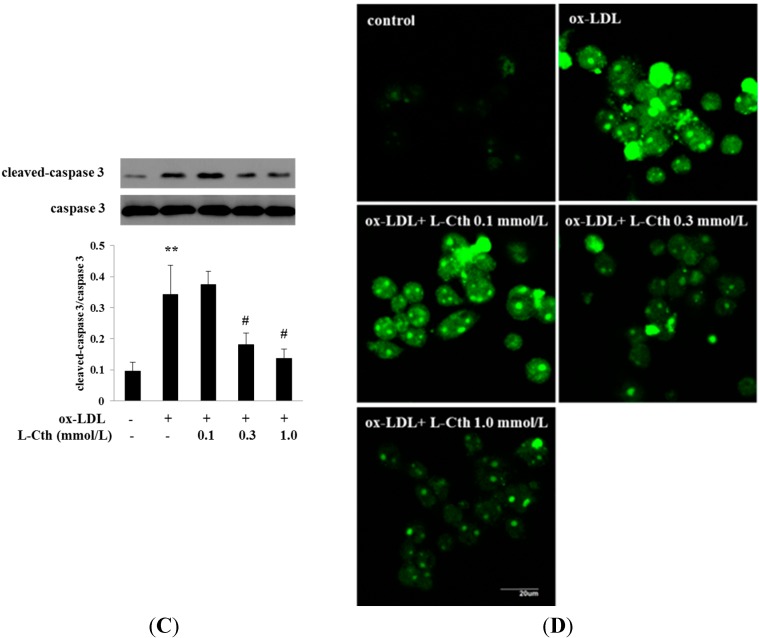
Changes in caspase-9 activities, cleavage of caspase-3, and cell apoptosis in human macrophages. (**A**) Caspase-9 activity detected by living cells caspase-9 Fluo-staining Kit; (**B**) Quantitative analysis of caspase-9 activities measured by cell caspase-9 assay; (**C**) Cleavage of caspase-3 analyzed by western blotting; (**D**) Cell apoptosis examined by TUNEL assay. l-Cth: l-cystathionine; ** *p* < 0.01 compared with control group; ^##^ *p* < 0.01 compared with ox-LDL group; ^#^ *p* < 0.05 compared with ox-LDL group. Data are presented as mean ± SD of three independent experiments performed in triplicate.

To study whether l-cystathionine could inhibit cell apoptosis induced by ox-LDL in human macrophages, we used fluorescence and colorimetric methods to measure caspase-9 activities. Data showed that fluorescence intensity was significantly strengthened in ox-LDL group compared with control group, suggesting increased caspase-9 activities, whereas 0.1 mmol/L l-cystathionine showed no significant impact on fluorescence intensity. However, fluorescence intensity was significantly weakened with treatment of 0.3 and 1.0 mmol/L l-cystathionine, suggesting the inhibition of caspase-9 activities (Figure 5A). Colorimetric methods also showed that compared with control group, ox-LDL markedly increased caspase-9 activities, but 0.1 mmol/L l-cystathionine exhibited no effects on caspase-9 activities. In contrast, administration of 0.3 and 1.0 mmol/L l-cystathionine significantly reduced caspase-9 activities (Figure 5B).

Then, western blotting was used to analyze the cleavage of caspase-3 protein. Results showed that ox-LDL significantly promoted caspase-3 cleavage. Pretreatment with 0.1 mmol/L l-cystathionine did not alter the expression of cleaved caspase-3 protein, while 0.3 and 1.0 mmol/L l-cystathionine significantly inhibited cleavage of caspase-3 (Figure 5C).

Finally, TdT-mediated dUTP nick-end labeling (TUNEL) assay was used to detect cell apoptosis. We found that fluorescence implying cell apoptosis was significantly enhanced in ox-LDL group compared with control group. No difference of fluorescence was found with 0.1 mmol/L l-cystathionine treatment compared with control cells. However, pretreatment with 0.3 and 1.0 mmol/L l-cystathionine significantly reduced fluorescence, indicating the suppression of cell apoptosis (Figure 5D).

## 3. Discussion

l-cystathionine locates at the hub in metabolism of sulfur-contained amino acids in mammals. Under the catalysis of methionine adenosyltransferase, methionine can be transformed to *S*-adenosylmethionine, then to *S*-adenosyl homocysteine after transmethylation, which is further hydrolyzed to homocysteine (Hcy). Hcy and serine synthesizes l-cystathionine under the catalysis of cystathionine-β-synthetase (CBS), while l-cystathionine decomposes into cysteine, α-ketonebutyric acid, and ammonium ions under the catalysis of cystathionine-γ-lyase [21]. Therefore, CBS is the key enzyme involved in l-cystathionine synthesis. In previous studies, it was generally considered that there was absence of l-cystathionine in adult cardiovascular system. However, in 1999, Quéré, *et al.* found extensive CBS expression in multiple tissues during the early embryonic development, particularly highest in nerve and heart tissues, while it was only detected in liver and brain of adults [22]. In 2003, Robert *et al.* proved that CBS transcription was detected in endocardial cells during the embryonic development of mouse, and CBS mRNA was also detected in aorta during the late development [23]; and in 2011, Sun *et al.* reported CBS expression in aorta and pulmonary artery rings of rats in the study examining the relaxation effects of hydrogen sulfide on aorta and pulmonary artery rings [24]. In terms of its function, Kouichirou *et al.* found that l-cystathionine could significantly reduce superoxide radicals generated by human leukocytes in a concentration-dependent manner *in vitro* [25], and another study shown that l-cystathionine was able to inhibit the apoptosis of U937 and HepG2 cells through preventing glutathione excretion [20]. All these results laid a foundation for us to investigate the regulatory role of l-cystathionine in human macrophage apoptosis.

**Figure 6:**
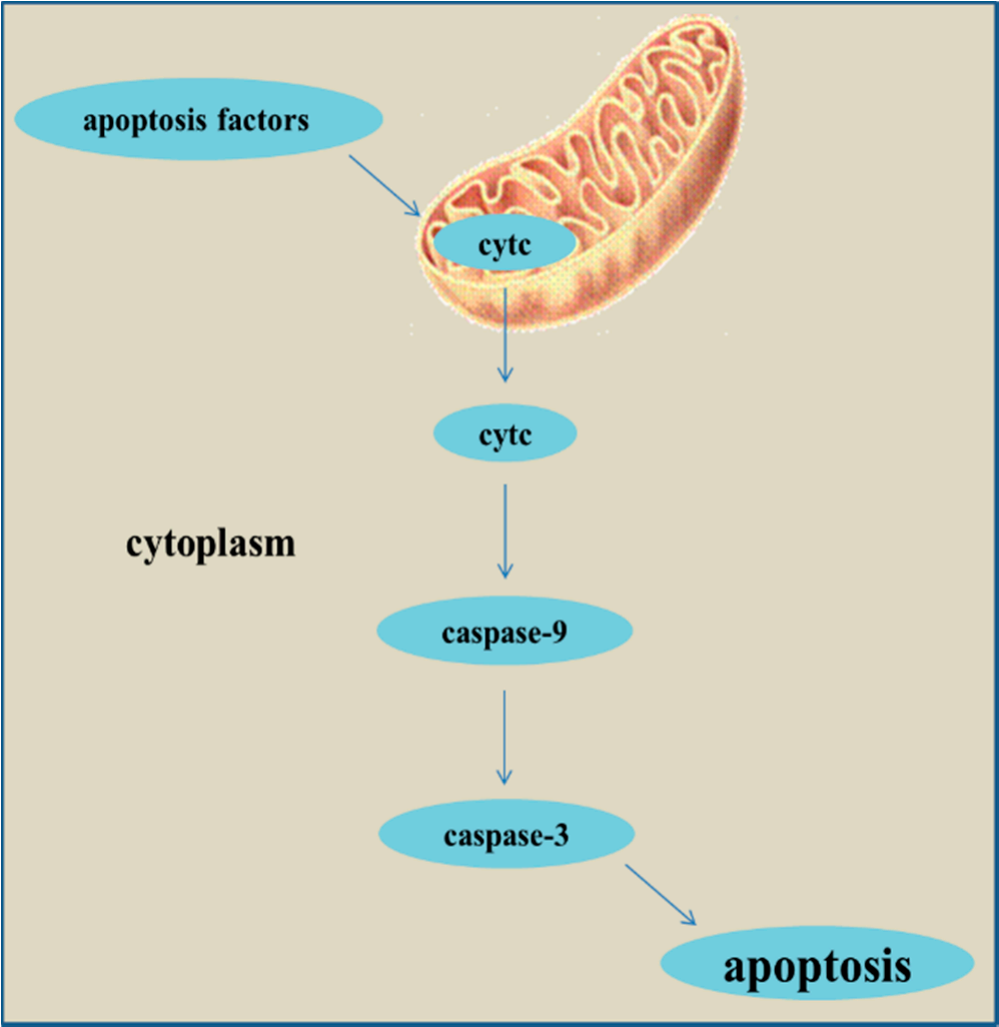
The pathway of mitochondria-mediated apoptosis.

Oxidative stress is the pathogenic factor of many cardiovascular diseases, and can induce cell apoptosis. Recently, more and more studies have proved that oxidative stress can lead to apoptosis through mitochondria-mediated pathway (cytc/caspase-9/caspase-3) and mitochondria-independent pathway [26]. MPTP is a non-specific calcium channel composed by inner and outer mitochondrial membranes, whose opening can be activated by external apoptosis factors. The persistent opening of MPTP results in a decline of mitochondrial membrane potential as well as cytc release. The release of cytc from mitochondria into cytoplasm is the key step in apoptosis [27] and cytoplasmic cytc activates caspase-9, which in turn activates caspase-3, thereby culminating in apoptosis [28,29] (Figure 6).

In this study, macrophages were differentiated from THP-1 monocytes induced by PMA under *in vitro* culture. Studies have shown that reactive oxidative stress (ROS) can activate casepase-3 and mitochondria-mediated macrophage apoptosis [30]. Firstly, we explored the effect of l-cystathionine on superoxide generation in human macrophages. We separately detected the superoxide expression in cells and mitochondria with DHE and MitoSOX probes, and the results showed that ox-LDL stimulated the production of superoxide in cells and mitochondria, which could be inhibited by 0.3 or 1.0 mmol/L l-cystathionine. Nonetheless, whether excessive superoxide production could activate mitochondria-mediated apoptosis pathway renders further investigation.

Once mitochondria were stimulated by pro-apoptotic signals, MPTP opened, and the mitochondrial membrane potential was decreased. Therefore, we explored the changes of mitochondrial membrane potential and MPTP. JC-1, a fluorescent probe widely used in the detection of mitochondrial membrane potential, could form aggregate in mitochondria matrix emitting red fluorescence under high mitochondrial membrane potential; and it remained monomer emitting green fluorescence under low mitochondrial membrane potential. JC-1 experiment showed that the mitochondrial membrane potential of human macrophages with ox-LDL stimulation was decreased significantly, and increased significantly after pretreatment with 0.3 or 1.0 mmol/L l-cystathionine. In MPTP fluorescence detection experiment, calcein-AM was used as probe, and calcein emitted green fluorescent after entering into mitochondria. Once MPTP opened, the released calcein can be quenched by cobalt ion, weakening the green fluorescence. The results showed that ox-LDL prompted the opening of MPTP in human macrophages, while pretreatment with 0.3 or 1.0 mmol/L l-cystathionine could inhibit the opening of MPTP.

Further, we explored whether l-cystathionine could prevent the release of cytc from mitochondria into cytoplasm. Western blot was used to detect the cytc protein expression in mitochondria and cytoplasm, respectively. Results showed that ox-LDL downregulated the cytc expression in mitochondria but upregulated it in cytoplasm, suggesting that ox-LDL promoted cytc release from mitochondria into cytoplasm. In contrast, compared with the ox-LDL group, 0.3 or 1.0 mmol/L l-cystathionine increased the cytc expression in mitochondria but reduced it in cytoplasm, suggesting that l-cystathionine could inhibit cytc release from mitochondria into cytoplasm.

The cytoplasmic cytc released from mitochondria could in turn activate caspase-9 and caspase-3, initiating apoptosis cascades. Thus, we hypothesized that l-cystathionine inhibited human macrophage apoptosis by suppressing caspase-9 and caspase-3 activation. First, we measured caspase-9 activity in human macrophages with fluorescence *in situ* staining and colorimetric methods. The results showed that ox-LDL significantly upregulated caspase-9 activity, while 0.3 or 1.0 mmol/L l-cystathionine significantly downregulated caspase-9 activity. Then, western blot was used to detect caspase-3 protein expression in human macrophages, and results showed that ox-LDL upregulated caspase-3 protein expression, while in the presence of 0.3 or 1.0 mmol/L l-cystathionine, the ox-LDL-induced upregulation of caspase-3 was abolished. Finally, TUNEL kit was used to detect the human macrophage apoptosis, and the results showed that ox-LDL induced human macrophage apoptosis, while pretreatment with 0.3 or 1.0 mmol/L l-cystathionine antagonized the ox-LDL-induced human macrophage apoptosis.

In conclusion, our results demonstrated that l-cystathionine could inhibit ox-LDL-induced mitochondria-mediated apoptosis initiated by excessive superoxide production, via increasing mitochondrial membrane potential, inhibiting MPTP opening, suppressing cytc release from mitochondria into cytoplasm as well as downregulating caspase-9 activities and caspase-3 protein expression. It is of great significance in revealing the inhibitory effects of l-cystathionine upon macrophage apoptosis in the late atherosclerosis diseases and providing a foundation for investigating the anti-apoptotic role of l-cystathionine in other apoptosis-related cardiovascular diseases. Of importance, l-cystathionine potentiates a novel approach for prevention and therapy of apoptosis-related cardiovascular diseases. In the future, further studies on human medical conditions with alterations in CBS expressions possibly affecting l-cystathionine metabolism in humans are needed.

## 4. Materials and Methods

### 4.1. THP-1 Monocytes Culture

THP-1 cell lines were purchased from ATCC Company (Washington, WA, USA). THP-1 cells with suspension growth were cultured in RMPI 1640 medium containing 10% fetal bovine serum, 100 U/mL streptomycin and 100 U/mL penicillin, placing in an incubator containing 5% CO_2_ at a constant temperature of 37 °C. Then, suspending cells were differentiated into adherent macrophages after induction with PMA for 24 h. The cells were cultured in serum-free RMPI 1640 medium to synchronize for 24 h before each experiment [31], and it was pretreated with l-cystathionine for 30 min then stimulated with ox-LDL for 6 h.

### 4.2. Detection of Mitochondrial Superoxide in Human Macrophages by MitoSOX Reagent

MitoSOX Red Mitochondrial Superoxide Indicator (Molecular Probes, Eugene, CA, USA) and MitoTracker Green FM (Molecular Probes) were used to detect the generation of mitochondrial superoxide. The human macrophages on slides were covered with MitoSOX reagent working solution, and incubated for 10 min at 37 °C, protecting from light. After washing with warm phosphate-buffered saline (PBS), the cells were incubated with MitoTracker probes for 20 min at 37 °C. The human macrophages were fixed in prewarmed 4% paraformaldehyde at room temperature for 20 min after washing with warm PBS three times. The slides were mounted by anti-fluorescence quencher (Beijing Zhongshan Golden Bridge Biotechnology Company, Beijing, China) after washing with PBS. Then the cells on slides were detected by a laser scanning confocal microscope. Red fluorescence indicated mitochondrial superoxide and green fluorescence indicated labeled mitochondria. Red and green fluorescence overlapping indicated the merged image.

### 4.3. Detection of Superoxide in Human Macrophages by Dihydroethidium (DHE)

Dihydroethidium (DHE) (Beyotime Institute of Biotechnology, Shanghai, China) was used to detect the generation of superoxide anion in human macrophages. The medium covering the cell was removed carefully, and the cells were incubated with DHE probes for 30 min at 37 °C in the dark after washing with warm PBS. Following washing gently three times with warm PBS, the cells were mounted in warm PBS for observing under a fluorescence microscope.

### 4.4. Measurement of Mitochondrial Membrane Potential in Human Macrophages by JC-1

For the purpose of measuring mitochondrial membrane potential, mitochondrial membrane potential assay kit with JC-1 (Beyotime Institute of Biotechnology, Shanghai, China) was used according to instructions [32]. Aggregation of JC-1 which presents red fluorescence in living cells when mitochondrial membrane potential is high, while monomer of JC-1 which presents green fluorescence in apoptotic cells when mitochondrial membrane potential is low. JC-1 working solution was added to the human macrophages on slides and incubated for 20 min at 37 °C in the dark. The cells were fixed in pre-warmed 4% paraformaldehyde at room temperature for 20 min after washing with ice-coldJC-1 buffer solution twice. The slides were subsequently mounted by anti-fluorescence quencher after washing with PBS three times. The cells on slides were examined under a laser scanning confocal microscope. Red fluorescence indicated high mitochondrial membrane potential and green fluorescence indicated low mitochondrial membrane potential. Red and green fluorescence overlapping indicated the merged image.

### 4.5. Measurement of MPTP Opening in Human Macrophages by Cell MPTP Assay Kit

MPTP opening was measured by cell MPTP assay kit (Genmed Scientific Inc., Alington, TX, USA). The medium covering the cell on slides was removed carefully, and gently rinsed with GENMED cleaning solution. Then, the human macrophages on slides were incubated with GENMED staining working solution for 20 min at 37 °C in the dark. The cells were fixed in pre-warmed 4% paraformaldehyde at room temperature for 20 min after rinsing with GENMED cleaning solution twice. Slides were mounted by anti-fluorescence quencher after washing with PBS three times. A laser scanning confocal microscope was used for analysis.

### 4.6. Detection of the Release of Cytc from the Mitochondrion into the Cytoplasm in Human Macrophages by Western Blot

Mitochondria isolation kit (Applygen Technologies Inc., Beijing, China) was used to extract mitochondrial and cytosolic protein [33], and all operations were conducted at 4 °C according to manufacture guidelines. The human macrophages were grinded with grinding pestles after resuspending in Mito Solution, and transferred to centrifuge tubes. The supernatant was moved into pre-cooled tubes after centrifugation (800× *g*) at 4 °C for 5 min. The supernatant was moved into new pre-cooled tubes after centrifugation (800× *g*) again at 4 °C for 5 min. Then the supernatant (cytosolic protein) was collected by new tubes after centrifugation (10,000× *g*) again at 4 °C for 10 min, and Mito Solution was added to the precipitate (containing mitochondrial protein) to wash mitochondria. Mito Solution was added to resuspend mitochondria after centrifugation (12,000× *g*) again at 4 °C for 10 min.

Mitochondrial and cytosolic protein concentration was measured by Bradford methods. Protein samples of 40 μg were separated in 10% SDS-PAGE and then electrically transferred onto nitrocellulose membrane. Then the membrane was blocked in 5% skim milk for 1 h and incubated with primary antibody against cytc (Beijing Zhongshan Golden Bridge Biotechnology Company, Beijing, China) at 4 °C overnight. Cytochrome c oxidase IV (COX IV) and β-tublin were used as the makers of mitochondrial and cytosolic protein, respectively. Horseradish peroxidase (HRP)-conjugated secondary antibody was added and incubated at room temperature for 1 h after washing. Immunoreactions were visualized by X-ray film exposure (Eastman Kodak Company, Rochester, NY, USA).

### 4.7. Measurement of Caspase-9 Activities in Human Macrophages by Fluorescence and Colorimetric Assay

Firstly, living cells caspase-9 Fluo-staining kit (Genmed Scientific Inc., Arlington, TX, USA) was used to detect caspase-9 activity. The medium covering the cell on slides was removed carefully, and gently washed with GENMED cleaning solution. Then slides were incubated with GENMED staining working solution for 20 min at room temperature in the dark. The cells were fixed in GENMED fixing solution at room temperature for 30 min after washing with GENMED cleaning solution two times. Then the slides were mounted by anti-fluorescence quencher after washing with PBS three times. A laser scanning confocal microscope was used for image analysis.

Then, cell caspase-9 assay kit (Genmed Scientific Inc., Arlington, TX, USA) was used to quantitative analysis ofcaspase-9 activities. The cells were scraped on ice with GENMED lysis solution after gently washing with GENMED cleaning solution. The supernatant was transferred into tubes after centrifugation (16,000× *g*) again at 4 °C for 5 min. Protein concentration was measured by Bradford method. The reagent and protein (40 μg) mixture was added in a 96-well plate and incubated for 2 h at 37 °C in the dark. Finally, the 96-well plate was read in a microplate reader (Bio-Rad, Hercules, CA, USA).

### 4.8. Cleavage of Caspase-3 in Human Macrophage Examined by Western Blotting

Extraction of total protein of human macrophages and measurement of protein concentration were described above. Protein samples of 40 μg were separated in 10% SDS-PAGE and then electrically transferred onto nitrocellulose membrane. Then the membrane was blocked in 5% skim milk for 1 h and incubated with primary antibody against cleaved caspase-3 and primary antibody against caspase-3 (Cell Signal Technology, Boston, MA, USA) at 4 °C overnight. HRP-conjugated secondary antibody was added and incubated at room temperature for 1 h after washing. Immunoreactions were visualized by X-ray film (Eastman Kodak Company, Rochester, NY, USA) exposure after the electrochemical luminescence (ECL).

### 4.9. Detection of Cell Apoptosis in Human Macrophages by TUNEL Assay

Cell apoptosis was detected using *in situ* cell death detection kit; and Fluorescein (Roche Applied Science; Mannheim, Germany) according to manufactures’ instructions [34]. The human macrophages on slides were fixed in 4% paraformaldehyde at room temperature for 30 min after washing with PBS. Then the cells were incubated with permeabilisation solution containing 0.1% Trition X-100 for 30 min at 37 °C after washing with PBS. Following washing twice with PBS, the slides were incubated with TUNEL reaction mixture in a humidified atmosphere for 60 min at 37 °C in the dark. Then the slides were mounted by anti-fluorescence quencher after washing with PBS three times. A laser scanning confocal microscope was used to examine cell apoptosis.

### 4.10. Statistical Analysis

SPSS 16.0 software was used for statistical analysis. One-way ANOVA analysis (ANOVA) was used for mean comparison among groups, and LSD test for comparison between two groups. *p* < 0.05 was considered statistically significant. Data are expressed as mean ± SD.

## References

[1] Libby P., Ridker P.M., Maseri A. (2002). Inflammation and atherosclerosis. Circulation.

[2] Seimon T., Tabas I. (2009). Mechanisms and consequences of macrophage apoptosis in atherosclerosis. J. Lipid Res..

[3] Tsukano H., Gotoh T., Endo M., Miyata K., Tazume H., Kadomatsu T., Yano M., Iwawaki T., Kohno K., Araki K. (2010). The endoplasmic reticulum stress-C/EBP homologous protein pathway-mediated apoptosis in macrophages contributes to the instability of atherosclerotic plaques. Arterioscler. Thromb. Vasc. Biol..

[4] Thorp E., Li G., Seimon T.A., Kuriakose G., Ron D., Tabas I. (2009). Reduced apoptosis and plaque necrosis in advanced atherosclerotic lesions of Apoe^−/−^ and LDLR^−/−^ mice lacking CHOP. Cell MeTable.

[5] Imanishi T., Akasaka T. (2013). Novel strategies to target inflammatory processes in atherosclerosis. Curr. Pharm. Des..

[6] Tabas I. (2005). Consequences and therapeutic implications of macrophage apoptosis in atherosclerosis: The importance of lesion stage and phagocytic efficiency. Arterioscler. Thromb. Vasc. Biol..

[7] Gillies L.A., Kuwana T. (2014). Apoptosis regulation at the mitochondrial outer membrane. J. Cell. Biochem..

[8] Skender B., Hofmanová J., Slavík J., Jelínková I., Machala M., Moyer M.P., Kozubík A., Hyršlová Vaculová A. (2014). DHA-mediated enhancement of TRAIL-induced apoptosis in colon cancer cells is associated with engagement of mitochondria and specific alterations in sphingolipid metabolism. Biochim. Biophys. Acta.

[9] Li Q., Sato E.F., Kira Y., Nishikawa M., Utsumi K., Inoue M. (2006). A possible cooperation of SOD1 and cytochrome c in mitochondria-dependent apoptosis. Free Radic. Biol. Med..

[10] Battaglia V., Salvi M., Toninello A. (2005). Oxidative stress is responsible for mitochondrial permeability transition induction by salicylate in liver mitochondria. J. Biol. Chem..

[11] Kumar S., Sitasawad S.L. (2009). *N*-acetylcysteine prevents glucose/glucose oxidase-induced oxidative stress, mitochondrial damage and apoptosis in H9c2 cells. Life Sci..

[12] Gaull G., Sturman J.A., Räihä N.C. (1972). Development of mammalian sulfur metabolism: Absence of cystathionase in human fetal tissues. Pediatr. Res..

[13] Zhang J., Zhang M., Ma D., Sugahara K., Kodama H. (1998). Metabolism of cystathionine, *N*-monoacetylcystathionine, perhydro-1,4-thiazepine-3,5-dicarboxylic acid, and cystathionineketimine in the liver and kidney of d,l-propargylglycine-treated rats. Metabolism.

[14] Aitken S.M., Kirsch J.F. (2005). The enzymology of cystathionine biosynthesis: Strategies for the control of substrate and reaction specificity. Arch. Biochem. Biophys..

[15] Zhang J., Sugahara K., Sagara Y., Fontana M., Duprè S., Kodama H. (1996). Effect of cystathionine ketimine on the stimulus coupled responses of neutrophils and their modulation by various protein kinase inhibitors. Biochem. Biophys. Res. Commun..

[16] Kodama H., Zhang J., Sugahara K. (2000). Novel priming compounds of cystathionine metabolites on superoxide generation in human neutrophils. Biochem. Biophys. Res. Commun..

[17] Kitamura Y., Kamisaki Y., Itoh T. (1989). Hepatoprotective effects of cystathionine against acetaminophen-induced necrosis. J. Pharmacol. Exp. Ther..

[18] Kwiecien N., Michalska M., Wlodek L. (2006). The selective effect of cystathionine on doxorubicin hepatotoxicity in tumor-bearing mice. Eur. J. Pharmacol..

[19] Maclean K.N., Greiner L.S., Evans J.R., Sood S.K., Lhotak S., Markham N.E., Stabler S.P., Allen R.H., Austin R.C., Balasubramaniam V. (2012). Cystathionine protects against endoplasmic reticulum stress-induced lipid accumulation, tissue injury, and apoptotic cell death. J. Biol. Chem..

[20] Ghibelli L., Fanelli C., Rotilio G., Lafavia E., Coppola S., Colussi C., Civitareale P., Ciriolo M.R. (1998). Rescue of cells from apoptosis by inhibition of active GSH extrusion. FASEB J..

[21] Klein C.E., Roberts B., Holcenberg J., Glode L.M. (1988). Cystathionine metabolism in neuroblastoma. Cancer.

[22] Quéré I., Paul V., Rouillac C., Janbon C., London J., Demaille J., Kamoun P., Dufier J.L., Abitbol M., Chassé J.F. (1999). Spatial and temporal expression of the cystathionine β-synthase gene during early human development. Biochem. Biophys. Res. Commun..

[23] Robert K., Vialard F., Thiery E., Toyama K., Sinet P.M., Janel N., London J. (2003). Expression of the cystathionine β-synthase (CBS) gene during mouse development and immunolocalization in adult brain. J. Histochem. Cytochem..

[24] Sun Y., Tang C.S., Jin H.F., Du J.B. (2011). The vasorelaxing effect of hydrogen sulfide on isolated rat aortic rings *versus* pulmonary artery rings. Acta Pharmacol. Sin..

[25] Kouichirou W., Yoshinori K., Kentaro N., Tadao I. (1996). Effect of cystathionine as a scavenger of superoxide generated from human leukocytes or derived from xanthine oxidase *in vitro*. Eur. J. Pharmacol..

[26] Sinha K., Das J., Pal P.B., Sil P.C. (2013). Oxidative stress: The mitochondria-dependent and mitochondria-independent pathways of apoptosis. Arch. Toxicol..

[27] Caroppi P., Sinibaldi F., Fiorucci L., Santucci R. (2009). Apoptosis and human diseases: Mitochondrion damage and lethal role of released cytochrome C as proapoptotic protein. Curr. Med. Chem..

[28] Li P., Nijhawan D., Budihardjo I., Srinivasula S.M., Ahmad M., Alnemri E.S., Wang X. (1997). Cytochrome c and dATP-dependent formation of Apaf-1/caspase-9 complex initiates an apoptotic protease cascade. Cell.

[29] Lin J.W., Chen J.T., Hong C.Y., Lin Y.L., Wang K.T., Yao C.J., Lai G.M., Chen R.M. (2012). Honokiol traverses the blood-brain barrier and induces apoptosis of neuroblastoma cells via an intrinsic bax-mitochondrion-cytochrome c-caspase protease pathway. Neurol. Oncol..

[30] Guo R., Su Y., Liu B., Li S., Zhou S., Xu Y. (2014). Resveratrol suppresses oxidised low-density lipoprotein-induced macrophage apoptosis through inhibition of intracellular reactive oxygen species generation, LOX-1, and the p38 MAPK pathway. Cell. Physiol. Biochem..

[31] Auwerx J. (1991). The human leukemia cell line, THP-1: A multifacetted model for the study of monocyte-macrophage differentiation. Experientia.

[32] Wang Z., Tang X., Li Y., Leu C., Guo L., Zheng X., Zhu D. (2008). 20-Hydroxyeicosatetraenoic acid inhibits the apoptotic responses in pulmonary artery smooth muscle cells. Eur. J. Pharmacol..

[33] Liu X.D., Sun H., Liu G.T. (2010). 5-Bromotetrandrine enhances the sensitivity of doxorubicin-induced apoptosis in intrinsic resistant human hepatic cancer Bel7402 cells. Cancer Lett..

[34] Gavrieli Y., Sherman Y., Ben-Sasson S.A. (1992). Identification of programmed cell death *in situ* via specific labeling of nuclear DNA fragmentation. J. Cell Biol..

